# Predictive Factors of Late Venous Aortocoronary Graft Failure: Ultrastructural Studies

**DOI:** 10.1371/journal.pone.0070628

**Published:** 2013-08-05

**Authors:** Bartlomiej Perek, Agnieszka Malinska, Sebastian Stefaniak, Danuta Ostalska-Nowicka, Marcin Misterski, Maciej Zabel, Anuj Suri, Michal Nowicki

**Affiliations:** 1 Department of Cardiac Surgery and Transplantology, University of Medical Science, Poznan, Poland; 2 Department of Histology and Embryology, University of Medical Science, Poznan, Poland; 3 Department of Pediatric Cardiology and Nephrology, University of Medical Science, Poznan, Poland; Istituto Clinico S. Ambrogio, Italy

## Abstract

**Background:**

Venous aortocoronary graft arterialization may precede a preterm occlusion in some coronary artery bypass grafting (CABG) patients. The aim of the present study was to identify ultrastructural variations in the saphenous vein wall that may have an impact on the development of venous graft disease in CABG patients.

**Methods:**

The study involved 365 consecutive patients with a mean age of 62.9±9.4 years who underwent isolated CABG. The thickness and area of the whole venous wall, the tunica intima, the tunica media and the adventitia and the number and shape (length, thickness and length/thickness ratio) of the nuclei in the medial smooth muscle cells nuclei in the distal saphenous vein segments were evaluated by ultrastructural studies. Patients were followed up for 41 to 50 months (mean 45.1±5.1). Saphenous vein graft patency was assessed by follow-up coronary angiography. Logistic regression models were used to identify independent risk factors for late graft failure.

**Results:**

In 71 patients significant lesions in the saphenous vein grafts were observed. The whole venous wall thickness (437.5 µm vs. 405.5 µm), tunica media thickness (257.2 µm vs. 211.5 µm), whole venous wall area (2.23 mm^2^ vs. 2.02 mm^2^) and tunica media area (1.09 mm^2^ vs. 0.93 mm^2^) were significantly larger for this group of patients than for those without graft disease. In the latter group more elongated smooth muscle cell nuclei (higher length/thickness ratio) were found in the tunica media of the saphenous vein segments. Thickening of the saphenous vein tunica media and chunky smooth muscle cell nuclei were identified as independent risk factors for graft disease development.

**Conclusions:**

Saphenous vein tunica media hypertrophy (resulting in wall thickening) and chunky smooth muscle cell nuclei might predict the development of venous graft disease.

## Introduction

Coronary artery bypass grafting (CABG) is the method of choice for the treatment of patients with advanced coronary artery disease (CAD). In majority of these patients, this procedure significantly improves prognosis and quality of life [1], [2]. Unfortunately, this clinical improvement may be only temporary. The recurrence of angina depends not only on the progression of atherosclerosis in the native coronary arteries but also on the patency rate of the conduits used for CABG [3], [4]. Although arterial grafts have higher late patency rate than venous conduits, autologous saphenous veins (SVs) are still the standard for vascular grafts due to their availability [5], [6], [7], [8].

The interposition of SV transplants into the arterial system exposes the venous wall to higher wall stretch forces and shear stress. These stresses may result in a series of the biological events within the venous wall that are called “venous grafts arterialization” [9], [10]. This process is characterized by intimal hyperplasia, thickening of the venous wall, migration of smooth muscle cells (SMCs) and the accumulation of extracellular matrix (ECM) in the tunica intima. Neointimal formation serves as the foundation for subsequent progressive graft atheroma, which eventually leads to graft occlusion months and years after surgery [11], [12].

The key factor in neointimal development is the change in the SMC phenotype from contractile to synthetic, followed by the abnormal proliferation and migration of these cells through the internal elastic lamina to the tunica intima [13], [14]. These cells respond to a variety of locally released mediators such as growth factors and cytokines [15], [16]. However, the responses of SMCs are not uniform in all venous aortocoronary bypass grafts recipients and the post-CABG treatment regimen may have an impact, but only to certain extent, on the SV transplant patency rate [5], [17]. There is currently little known about why SV segments with similar intraoperative macroscopic morphologies and harvested in the same manner, differ in early and late patency rates.

Thus, the aim of present study was to identify variations in the SV wall ultrastructure and relate them to the venous graft failure observed in CABG patients.

## Patients and Methods

### Study Population

All consecutive patients who underwent isolated non-emergent CABG with at least one venous aortocoronary bypass graft between July 2008 and May 2009 were considered for enrolment in this study. The exclusion criteria included the following: the necessity of an emergent operation due to cardiogenic shock, acute complications of percutaneous coronary interventions (PCIs) and lower extremity vein pathologies (e.g., varices, venous thrombo-embolic disease, trauma, inflammation etc). SVs were also not harvested from patients with previously diagnosed with severe *atherosclerosis obliterans* due to the potential negative impact on wound healing. The preoperative data are outlined in Table 1.

**Table 1:** Selected pre- and intraoperative clinical data.

Variable^a^	Patients n = 365
Age [mean±sd]	62.9±9.4
Gender [M/F]	272 (74.5)/93 (25.5)
BMI	29.3±4.1
Obesity (BMI>30) [n(%)]	142 (38.9)
Arterial hypertension	264 (72.3)
Diabetes mellitus	162 (44.4)
Diabetes mellitus treated with insulin	72 (19.7)
Hyperlipidemia	153 (41.9)
Smoking^b^	179 (48.2)
Family history of CAD	131 (35.9)
Stable angina	302 (82.7)
Unstable angina	63 (17.3)
History of infarct	217 (59.5)
Previous PCI	104 (28.5)
Left main disease	135 (37.0)
Three vessels disease	161 (44.1)
Two vessels disease	51 (14.0)
One vessel disease	18 (4.9)
LVEF	0.53±0.11
CCAB/OPCAB	177 (48.5)/188 (51.5)
Number of grafts/pt	2.7±0.5
Number of grafts [distal anastomoses]	990
Arterial (LITA, RITA, RA)	386
Venou	604
Complete revascularization^c^ [n(%)]	257 (70.4)

a Categorical variables are presented as the number (%) and continuous variables as the mean ± standard deviation;

b the term “smoking” comprises active smokers and the individuals who had given up smoking within one year before surgery;

c all severely stenotic or occluded native coronary arteries were bypassed.

Abbreviations: BMI = body mass index; CAD = coronary artery disease; CCAB = conventional coronary artery bypass (in the extracorporeal circulation); LITA = left internal thoracic artery; LVEF = left ventricular ejection fraction (by echocardiography); OPCAB = off-pump coronary artery bypass; PCI = percutaneous coronary intervention; RA = radial artery; RITA = right internal thoracic artery.

### Ethics Statement

The Institutional Review Board at the Poznan University of Medical Sciences approved the study protocol (Approval No.1201/08) and informed written consent was obtained from each study participant.

### Operation and Sample Collection

Some of the operations were performed in the cardiopulmonary bypass (CCAB, conventional coronary artery bypass) and the others off-pump (OPCAB, off-pump coronary artery bypass) (Table 1).

SVs were obtained through full-length thigh incision over its course. Meticulous surgical technique to prevent extensive dilation of the harvested vein and low-intensity electrocautery were employed.

In all patients the most distal portions of the harvested SV segments (approximately 2 cm long) were taken for histological studies. These segments were carefully rinsed with 0.9% NaCl at room temperature and then slightly dilated and split into two equal parts. The first part was fixed in the freshly prepared Bouin’s solution for histological examination under a light microscope and the second one was fixed in 2.5% glutaraldehyde in phosphate buffer (0.05 M, pH 7.4) for the electron microscopy study.

### Morphometric Analysis

Fixed SV segments were embedded in paraffin blocks using a routine procedure and cut into 5-µm sections on a semi-automatic rotary microtome (Leica RM 2145, Leica Microsystems, Nussloch, Germany). Every fifth SV section was stained with hematoxylin and eosin (H&E) and examined using a AxioImager Z.1 light microscope (Carl Zeiss MicroImaging GmbH, Göttingen, Germany) at a magnification between 50 and 400x. Representative images of each SV segment were captured with an attached AxioCam MRc5 digital camera (Carl Zeiss) and then analyzed using of AxioVisionLE for Windows ver. 4.8.2. (Carl Zeiss).

The thicknesses of the whole venous wall (WallTh) and all its layers (intima (IntTh), media (MedTh) and adventitia (AdvTh)) were measured at eight areas throughout the vessel circumference at 50×magnification. The average values of at least 40 measurements (5 to 6 slides and 8 measurements per one slide) were then calculated. Additionally, the cross-sectional areas of the whole venous wall (WallAr) and each of its layers (IntAr, MedAr and AdvAr, respectively) were calculated for 5 slides per SV segment. The correlations between WallAr or WallTh and all the thickness or area of each SV layer were also estimated. Two additional indices, the relative tunica media thickness (%MedTh = MedTh/WallTh) and area (%MedAr = MedAr/WallAr), were also calculated. Subsequently, the number of SMC nuclei within a 50×50 µm square was counted at 400×magnification and then total number of SMC nuclei in the cross-sections was estimated.

Additionally, the shape (length, thickness and length/thickness ratio) of 10 representative SMC nuclei in every vessel specimen (usually 6 specimens per patient) was assessed at 400×magnification. The associations between the shape of the SMC nuclei and other morphometric variables such as WallTh, WallAr, MedTh and MedAr were analyzed.

### Transmission Electron Microscopic Study Protocol

Material fixed in 2.5% glutaraldehyde in phosphate buffer (0.05 M, pH 7.4) was post-fixed in 1% OsO_4_ in the same buffer, dehydrated and embedded in Araldite (Polysciences Europe, Germany). Semithin and ultrathin sections were cut on a Leica Ultracut UCT (Leica Microsystems, Nussloch, Germany). The 0.3-µm-thick sections were stained with toluidine blue and examined under an Olympus BX50 light microscope (Olympus Optical Europe, Germany). Ultrathin sections were mounted on mesh nickel grids and then counterstained with uranyl acetate and lead citrate. These sections were examined using a JEM-1010 (JOEL, Tokyo, Japan) transmission electron microscope.

### Evaluation of Coronary Outcomes

All patients were followed-up systematically in the outpatient clinic. Unless contraindicated, the patients were routinely treated medically with statins and acetylsalicylic acid/clopidogrel. In addition, all patients were educated on how to control risk factors for atherosclerosis progression.

The post-discharge coronary status according to the Canadian Cardiovascular Society (CCS) classification was evaluated at least twice: after the completion of the postoperative rehabilitation program and at the end of the follow-up period. A four-week CCS class served as a baseline for the final follow-up CAD evaluation.

In patients with acute coronary syndromes (ACSs) (unstable angina, myocardial infarction with (STEMI) or without ST segment elevation (NSTEMI)) or manifestations of CAD progression (according to the CCS classification), coronary angiography was performed emergently and then again at the end of follow-up period. In subjects without clinical progression of CAD but with insulin-treated diabetes (DM), coronary angiography was usually performed twice, 24 months after surgery and at the end of the follow-up period. Coronary angiographic was also performed at the end of the follow-up period in 105 individuals without either clinical CAD progression or insulin-treated DM but with other preexisting risk factors for atherosclerosis progression, such as hypertension, hyperlipidemia, obesity, active smoking and family history of CAD. In summary, 235 subjects underwent invasive coronary angiography. All aortocoronary grafts and the native arteries were analyzed. Hemodynamically significant lesions were defined as a lumen restriction exceeding 70%.

### Histological Findings for Patients with Venous Graft Disease

Selected clinical data and the prevalences of low (below the 25^th^ percentile) and high (exceeding the 75^th^ percentile) values of the morphometric variables were compared between the groups of patients with (SVGD (+)) and without SV graft disease (SVGD (−)) according to the follow-up coronary angiography.

### Data Management and Statistical Analysis

The Shapiro-Wilk W test for normality was performed for all continuous variables. When the values normally distributed, they were expressed as the means ± standard deviations and then compared using Student’s unpaired t test. Additionally, the 25^th^ and 75^th^ percentiles for the selected morphometric variables were calculated. Non-normally distributed continuous variables were expressed as the median (25^th^ percentile; 75^th^ percentile) and compared with the Mann-Whitney U test. Categorical data were expressed as numbers (*n*) and percentages (%) and were compared with Pearson χ^2^ test. A P value <0.05 was considered statistically significant. The correlations between morphometric variables were tested using linear Pearson’s r correlation coefficients. The Pearson correlation coefficients were interpreted using the scale provided by Salkin, where an r between 0.8 and 1.0 (or −0.8 and −1.0) is defined as very strong, between 0.6 and 0.8– as strong, between 0.4 and 0.6– as moderate, between 0.2 and 0.4– as weak and between 0.0 and 0.2– as very weak or no relationship [18]. The probabilities of survival and a freedom from clinical CAD deterioration among the survivors were estimated using the Kaplan-Meier method.

Logistic regression analysis was performed to identify histopathological predictors of post-discharge CAD deterioration related to the development of severe venous graft disease. Morphological data were screened using a univariate model, and then the variables with a P value of <0.2 were entered into a multivariate logistic regression model. Variables revealed to be significant (P<0.05) in the latter model were considered to be independent predictors of accelerated venous aortocoronary graft failure. The following variables were entered into the logistic regression model: high (H; exceeding the 75^th^ percentile) and low (L; below the 25^th^ percentile) values of the variables used in the morphometric analysis including MedAr and MedTh and absolute number of SMC nuclei in the SV cross-sections swell as well as their shape (represented as the nuclei length/thickness ratio). Additional multivariate logistic regression models were created to identify clinical predictors of tunica media hypertrophy (first model) or the presence of chunky SMC nuclei (second model). Hypertrophy was defined as a MedTh exceeding the 75^th^ percentile, and chunky SMC nuclei were defined as nuclei for with the length/thickness ratio was below the 25^th^ percentile. Variables including an age >70 years, gender, obesity, arterial hypertension, diabetes treated with insulin, hyperlipidemia, family history and unstable angina were entered into additional logistic regression models.

All statistical analyses were performed using Statistica 9.0 for Windows (StatSoft, Inc., Tulsa, OK, USA).

## Results

### Clinical Results and Coronary Outcomes

This prospective study involved 365 consecutive patients (272 males and 93 females) with a mean age of 62.9±9.4 (ranged from 31 to 85 years). Four patients (1.1%) died in the early postoperative period (defined as 30 days after surgery) due to postcardiotomy low cardiac output syndrome (n = 3) and gastrointestinal complications (n = 1). During the follow-up period which lasted from 41 to 50 months (average 45.1±5.1 months) another seven deaths were noted: 3 due to cardiac reasons (ST segment elevation myocardial infarction (n = 1) and progressive heart failure (n = 2)) and 4 due to non-cardiac reasons (neurological (n = 1), malignancies (n = 2), multi-organ trauma (n = 1)). The survival probability following CABG in our series was 98.6±0.6% at 1 year, 97.8±0.7% at 2 years and 96.9±0.9% at 4 years after the primary surgery.

All aforementioned individuals and those (n = 5) lost during follow-up were not included in the analysis of the late CAD status because clinical evaluation was not possible. Perioperative myocardial infarction was diagnosed in 8 patients. This diagnosis was established according to the latest European Society of Cardiology definition of myocardial infarction related to CABG (type 5) [19]. In total, 341 patients underwent late coronary status evaluation, including 235 patients who underwent invasive coronary angiography.

A significant improvement in the CCS status was noted at the time of the completion of the postoperative rehabilitation program (P<0.001, CCS status before vs. four weeks after surgery). Before surgery, only 29 (7.9%) patients had no or mild CAD symptoms (classes 0 and I according to the CCS classification), and this number increased to 338 (96.0%) soon after operation. Although the CABG outcome deteriorated slightly over time during the follow-up period, 288 (84.4%) individuals still exhibited signs of significant benefit from surgery in terms of coronary symptom alleviation (CCS classes 0 and I) at the last examination.

**Figure 1:**
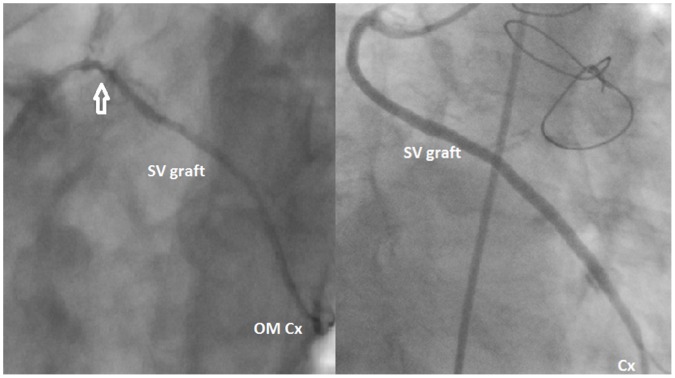
Follow-up coronary angiography. Fig. 1a. SVGD (⇑) in the graft implanted to the obtuse marginal branch of the circumflex artery in a 58-year old man admitted to a regional hospital due to unstable angina 27 months after surgery. The morphometric analysis of this SV segment obtained intraoperatively (see Figure 2a) revealed marked hypertrophy of the SV wall (WallTh 475.8 µm and WallAr 2.58 mm^2^) and its media (MedTh 329.2 µm and MedAr 1.43 mm^2^). The mean length/width index of the representative medial SMC nuclei was 5.7. Fig. 1b. Angiography of a normal SV aortocoronary bypass graft to the circumflex artery of a 75-year-old female patient without clinical symptoms of CAD deterioration performed 47 months after CABG. The results of the morphometric study (see Figure 2b) were as follows: WallTh 329.2µm, WallAr 1.55 mm^2^, MedTh 199.8 µm, MedAr 0.60 mm^2^. The SMC nuclei were spindle-shaped with a mean length/width index of 10.1. Abbreviations: Cx – circumflex artery; OMCx – obtuse marginal branch of the circumflex artery; SVgraft – saphenous vein bypass graft.

During the post-discharge follow-up, 24 patients were readmitted to the hospital due to ACS (STEMI in 6, NSTEMI in 14 and unstable angina in 4), and post-discharge angina recurrence or exaggeration according to the CCS classification was noted in 47 individuals. In the aforementioned groups of patients culprit lesions were identified in the SV grafts in 62 cases (87.3%) (Figure 1a). In 9 patients without CAD clinical progression but with insulin-treated DM, severe SV graft disease was observed. In asymptomatic patients without DM, all grafts both arterial and venous, were patent without lesions that reduced the graft’s diameter or compromised flow (Figure 1b). The detailed coronary angiography findings are summarized in Table 2.

**Table 2:** Findings of the follow-up coronary angiography.

	Location of culprit lesions			
Indication for angiography	IsolatedSVGD	SVGD and nativearteries^1^	Isolated arterialgrafts	Native arteries (without grafts)
ACS [n = 24]	18	3	1	2
CAD deterioration [n = 47]	26	15	2	4
DM treated with insulin [n = 59]without CAD deterioration	4	5	0	5
Asymptomatic individuals [n = 80]	0	0	0	1
	48	23	3	12

1 only lesions located in ungrafted coronary arteries or in segments of grafted arteries but distal to the sites of anastomoses with aortocoronary grafts.

Abbreviations: ACS = acute coronary syndrome; CAD = coronary artery disease; DM = diabetes mellitus; SVGD = saphenous vein graft disease.

The probability of survival without CAD progression calculated by Kaplan-Meier method was 99.1±0.5% at 1 year, 94.1±1.3% at 2 years and 77.2±2.7% at 4 years after surgery.

### Comparison of SVGD (+) and SVGD (−) Patients

A comparison of the selected preoperative data revealed that the SVGD (+) patients were more often male (86% vs. 73%; P<0.05) and were significantly younger (58.3 years vs. 64.1 years; P<0.001) than the SVGD (−) individuals (Table 3). More than one-third (39%) of the SVGD (+) subjects were 50 years old or younger, whereas in individuals in the SVGD (−) accounted for only 11% of SVGD (−) patients. One-fourth of the SVGD (+) patients were active smokers, and half of them had family history of CAD (P<0.05; group SVGD (+) vs. SVGD (−)). In addition, the preoperative laboratory examinations revealed higher white blood cell counts (WBC) and platelet counts (PLT) as well as increased concentrations of pro-atherogenic lipoproteins (cholesterol and the LDL fraction). Surprisingly, the SVGD (+) patients had significantly better preoperative systolic left ventricular performance estimated based on the left ventricular ejection fraction (LVEF) than the SVGD (−) individuals (0.62±0.08 vs. 0.56±0.10; P<0.001). Additionally, the SVGD (−) subjects underwent OPCAB procedures more often than the SVGD (+) patients did. Although more SVGD (−) individuals (77%; n = 207) than SVGD (+) (63%; n = 45) individuals underwent complete surgical revascularization, the difference between examined groups with respect to the average number of grafts per patient did not reach statistical significance.

**Table 3:** Comparison of the selected pre- and intraoperative variables between SVGD (+) and SVGD (−) patients.

Variable^a^	Group SVGD (+) n = 71	Group SVGD (−) n = 71	*P* value
Age [years]	58.3±8.4	64.1±8.5	<0.001
Age ≤ 50 years	27 (38.6)	30 (11.1)	<0.001
Age >70 years	10 (14.3)	72 (26.7)	0.015
Gender male/female	60 (85.7)/10 (14.3)	198 (73.3)/72 (26.7)	0.015
BMI [kg/m^2^]	29.9±4.4	28.9±4.0	0.111 ns
Obesity (BMI >30)	31 (44.3)	109 (40.4)	0.559 ns
Preoperative unstable angina	10 (14.3)	48 (17.8)	0.470 ns
Previous myocardial infarct	44 (62.9)	174 (64.4)	0.081 ns
History of PCI	13 (18.6)	88 (32.6)	0.012
Arterial hypertension	54 (77.1)	198 (73.3)	0.297 ns
Diabetes mellitus	31 (44.3)	122 (45.9)	0.815 ns
Diabetes treated with insulin	15 (21.4)	50 (18.5)	0.644 ns
Hyperlipidemia	25 (35.7)	118 (43.7)	0.376 ns
Active smoking	20 (28.6)	41 (15.2)	0.025
Family history of CAD	35 (50.0)	90 (33.7)	0.017
COPD	9 (12.9)	8 (3.0)	0.020
WBC [10e9/L]	7.87±1.15	7.19±1.02	0.036
RBC [10e12/L]	4.82±0.51	4.77±0.54	0.096 ns
PLT [10e9/L]	243.8±42.6	211.9±49.1	0.042
Total cholesterol [mmol/L]	5.07±1.04	4.68±1.19	0.010
LDL cholesterol [mmol/L]	3.25±0.97	2.73±0.75	0.020
HDL cholesterol [mmol/L]	1.24±0.25	1.19±0.25	0.105 ns
Triglycerides [mmol/L]	1.78±0.95	1.55±0.86	0.079 ns
Left main disease	22 (31.4)	107 (39.6)	0.198 ns
Three vessels disease	33 (47.1)	131 (48.5)	0.643 ns
LVEF	0.62±0.08	0.56±0.10	<0.001
LVEF <0.40	4 (5.6)	22 (8.1)	0.067 ns
CCAB/OPCAB	43 (60.6)/28 (39.4)	119 (44.1)/151 (55.9)	0.014
Number of grafts/patient	2.6±0.5	2.8±0.6	0.078 ns
Complete revascularization b	45 (63.4)	207 (76.7)	0.033

a Categorical variables are presented as the number (%) and continuous variables as the mean ± standard deviation;

b all severely stenotic or occluded native coronary arteries were grafted.

Abbreviations: BMI = body mass index; CAD = coronary artery disease; CCAB = conventional coronary artery bypass (in the extracorporeal circulation); COPD = chronic obstructive pulmonary disease; HDL = high density lipoproteins; LDL = low density lipoproteins; LVEF = left ventricular ejection fraction (by echocardiography); OPCAB = off-pump coronary artery bypass; PCI = percutaneous coronary intervention; PLT = platelet count; RBC = red blood cell count; SVGD = saphenous vein graft disease; WBC = white blood cell (leucocyte) count.

**Figure 2:**
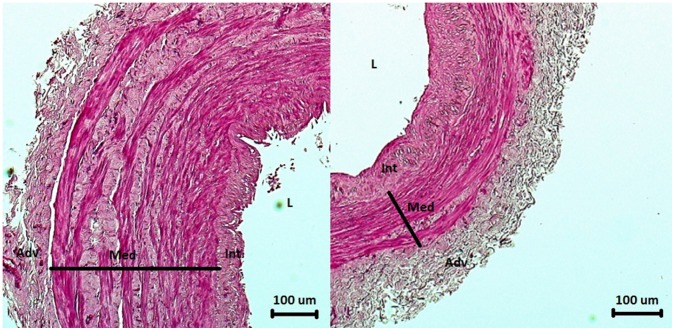
Ultrastructural appearances of the SV segments (H+E). Fig. 2a. An SV segment obtained from a 58-year-old man who was admitted to a regional hospital 27 months after surgery due to unstable angina. Coronary angiography revealed a significant lesion in the venous graft to the right coronary artery (see Figure 1a). Note the hypertrophic tunica media. Fig. 2b. An SV segment obtained from a 71-year-old male patient with no clinical symptoms of CAD progression and normal SV aortocoronary grafts (see Figure 1b). WallTh and MedTh were below the 25^th^ percentile. Abbreviations: Adv – adventitia; Int – tunica intima; L – lumen; Med – tunica media.

Detailed analysis of the morphometric variables revealed a number of significant differences between SVGD (+) and SVGD (−) patients (Table 4). In the SVGD (+) subjects, the entire wall (WallTh) (437.5 µm vs. 405.5 µm; P = 0.001) and the tunica media (MedTh) (257.2 µm vs. 211.5 µm; P<0.001) were significantly thicker than in the SVGD (−) study participants. Moreover, WallAr and MedAr were significantly larger in the SVGD (+) patients (2.23 mm^2^ vs. 2.02 mm^2^ and 1.09 mm^2^ vs. 0.93 mm^2^, respectively) than in the SVGD (−) patients (Figure 2b and 2b). The statistical analysis revealed strong correlations not only between MedAr and WallAr (r = 0.680; P<0.001), and MedTh and WallTh (r = 0.713; P<0.001) but also between MedTh and WallAr (r = 0.626; P<0.001), and MedAr and WallTh (r = 0.596; P<0.001). No associations between either the thickness or the area of other wall layers and wither WallTh and WallAr were found.

**Table 4:** Thickness and area of the whole venous wall and its layers in all patients enrolled in the study and in patients with and without a diagnosis of SVGD (SVGD (+) vs. SVGD (−)).

Variables	Overall^a^, n = 365	SVGD (−)^b^, n = 270	SVGD (+), n = 71	P value
WallTh [µm]	411.6±83.8 (359.1; 469.3)	405.5±82.8	437.5±89.2	0.001
L Wall Th		76 (28)	10 (14)	0.008
H Wall Th		55 (19)	31 (44)	<0.001
WallAr [mm^2^]	2.16±0.72 (1.56; 2.76)	2.02±0.76	2.23±0.57	0.027
L Wall Ar		79 (29)	6 (8)	<0.001
H Wall Ar		68 (25)	18 (25)	0.110 ns
IntTh [µm]	55.7±23.8 (39.7; 68.5)	55.3±23.5	56.7±25.5	0.674 ns
L Int Th		65 (24)	20 (28)	0.322 ns
H Int Th		69 (26)	16 (23)	0.975 ns
IntAr [mm^2^]	0.27±0.16 (0.14; 0.37)	0.28±0.17	0.26±0.11	0.326 ns
L Int Ar		69 (26)	17 (24)	0.654 ns
H Int Ar		71 (26)	15 (21)	0.068 ns
MedTh [µm]	221.1±56.9 (177.2; 259.0)	211.5±53.5	257.2±58.1	<0.001
L Med Th		78 (29)	7 (10)	<0.001
H Med Th		47 (17)	38 (54)	<0.001
MedAr [mm^2^]	0.98±0.41 (0.66; 1.24)	0.93±0.43	1.09±0.28	0.043
L MedAr		79 (29)	7 (10)	<0.001
H MedAr		63 (23)	22 (31)	0.044
AdvTh [µm]	161.8±45.2 (127.2; 193.9)	162.4±44.2	159.1±47.6	0.141 ns
L AdvTh		64 (24)	21 (30)	0.388 ns
H AdvTh		63 (23)	23 (32)	0.120 ns
AdvAr [mm^2^]	0.90±0.33 (0.66; 1.12)	0.90±0.34	0.89±0.33	0.586 ns
L AdvAr		67 (25)	18 (29)	0.607 ns
H AdvAr		71 (27)	15 (23)	0.459 ns
%MedTh	53.7±8.2 (48.7; 59.0)	52.2±7.6	59.3±7.6	<0.001
L %MedTh		77 (29)	7 (10)	<0.001
H %MedTh		39 (15)	37 (52)	<0.001
%MedAr	44.8±8.0 (39.1; 50.2)	43.4±7.9	47.7±7.5	<0.001
L %MedAr		79 (29)	5 (7)	>0.001
H %MedTh		57 (21)	28 (39)	0.008

a Variables are expressed as the mean ± standard deviation with the 25^th^ and the 75^th^ percentiles in brackets.

b Variables are expressed as the mean ± standard deviation and as numbers (*n*) with percentages (%) for the values below the 25^th^ percentile (L) and exceeding the 75^th^ percentile (H).

NOTICE: The sum of the SVGD (+) and SVGD (−) patients is lower than the total number of patients enrolled in the study because some study participants did not undergo late coronary evaluation (see the text).

Abbreviations: %MedAr = relative tunica media area (MedAr/WallAr); %MedTh = relative tunica media thickness; AdvAr = adventitia area; AdvTh = adventitia thickness; IntAr = tunica intima area; IntTh = tunica intima thickness; MedAr = tunica media area; MedTh = tunica media thickness; SVGD = saphenous vein graft disease; WallAr = entire area of SV wall; WallTh = thickness of the SV wall.

More SMC nuclei were found in the tunica media in the cross sections of SV segments harvested from the SVGD (+) patients (Table 5). The medial SMC nuclei in the SVGD (−) patients were significantly longer, and thinner and, consequently, had a higher length to thickness ratio than the medial SMC nuclei in the SV transplants from SVGD (+) patients.

**Table 5:** A comparison of the data for the number and shape of SMC nuclei between SVGD (+) and SVGD (−) subjects.

Variables	Overall^a^, n = 365	SVGD (−)^b^, n = 270	SVGD (+), n = 71	P value
Absolute number of medial SMCs [n]	3091±1270 (2140; 3840)	3020±1330	3404±975	0.011
		L: 80 (30)	L: 5 (7)	<0.001
		H: 65 (24)	H: 20 (28)	0.156 ns
Thickness [µm]	1.75±0.20 (1.63; 1.86)	1.72±0.20	1.85±0.17	<0.001
		L: 78 (29)	L: 7 (10)	0.001
		H: 60 (22)	H: 24 (34)	<0.001
Length [µm]	14.23±1.47 (13.24; 15.22)	14.36±1.48	13.63±1.27	<0.001
		L: 60 (22)	L: 25 (35)	0.046
		H: 76 (28)	H: 8 (11)	<0.001
Area [mm^2^]	22.96±3.07 (20.89; 24.55)	22.98±3.13	22.76±2.79	0.580 ns
		L: 64 (24)	L: 20 (28)	0.359 ns
		H: 63 (23)	H: 21 (29)	0.273 ns
Index [length/thickness]	8.36±1.08 (7.73; 9.17)	8.50±1.10	7.79±0.80	<0.001
		L: 56 (21)	L: 26 (37)	0.009
		H: 82 (30)	H: 2 (3)	<0.001

a Variables are expressed as the mean ± standard deviation with the 25^th^ and the 75^th^ percentiles in brackets.

b Variables are expressed as the mean ± standard deviation and as numbers (*n*) with percentages (%) for the values below the 25^th^ percentile (L) and exceeding the 75^th^ percentile (H).

NOTICE: The sum of the SVGD (+) and SVGD (−) patients is lower than the total number of patients enrolled in the study because some study participants did not undergo late coronary evaluation (see the text).

Abbreviations: SMC = smooth muscle cell; SVGD = saphenous vein graft disease.

**Figure 3:**
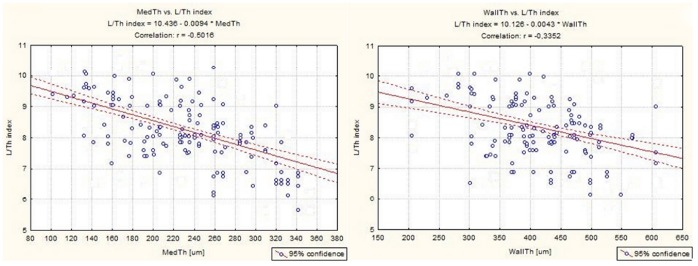
Association between SV wall or tunica media thickness and the SMC nuclei shape. The moderate negative correlations were found between the shape of the SMC nuclei (L/Th index, length/thickness index) and both MedTh (Fig. 3a) and WallTh (Fig. 3b).

In the statistical analysis, moderate negative correlations between MedTh and the SMC nuclei length-to- thickness ratio (r =  - 0.502; P<0.001) (Figure 3a) and between WallTh and the SMC nuclei length-to-hickness ratio (r =  - 0.335; P<0.001) (Figure 3b) were found.

**Figure 4:**
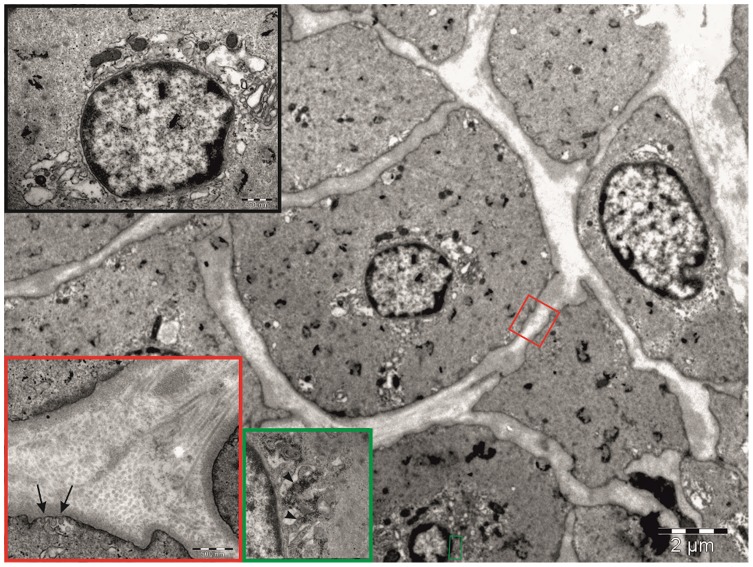
TEM analysis of a graft from an SVGD (+) patient. Electron micrograph of a cross section through the tunica media layer of the saphenous vein obtained from an SVGD (+) patient. Characteristic nuclei with heterochromatin are present at the periphery. The organelles are concentrated in proximity to the nucleus (black inset), and the extracellular matrix attached to densely packed and organized collagen fibers (red inset). Note the small invaginations of cell membrane forming caveolae (arrows) and the numerous glycogen deposits (arrowheads, green inset).

**Figure 5:**
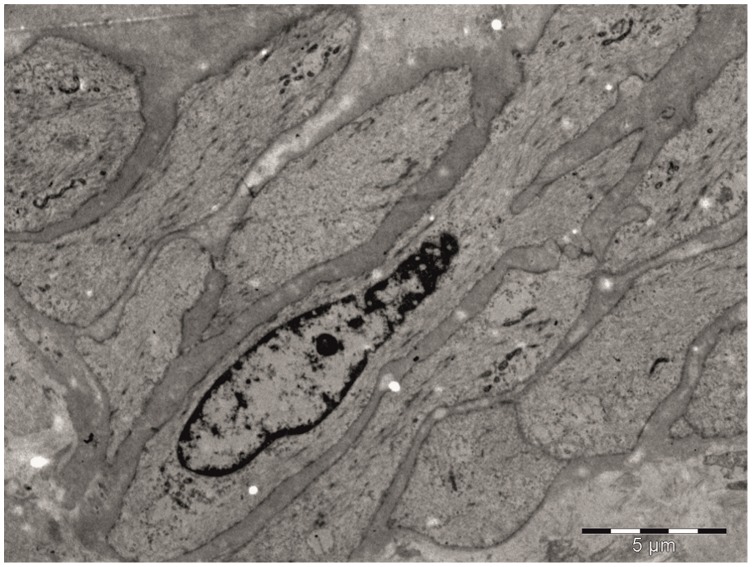
TEM analysis of a graft from an SVGD (−) patient. Electron micrograph showing the tunica media of the saphenous vein obtained from an SVGD (−) patient. Smooth muscle cells with typical organization for this layer were observed. Note, that the cytoplasm is poorly equipped with organelles. Only a small number of mitochondria were observed.

Transmission electron microscopy study revealed that in the SVGD (+) subjects SMCs were equipped with well-developed rough endoplasmic reticulum and Golgi complexes (Figure 4). The cytoplasm was filled with numerous glycogen deposits (Figure 4). In the SVGD (+) patients, well-packed and ordered collagen fibers were observed. In the SVGD (−) patients the majority of the SMCs had centrally located nuclei and a markedly reduced number of intracellular synthetic structures (Figure 5).

### Results of the Logistic Regression Models (Table 6)

The logistic regression model based on histopathological features showed that a hypertrophic tnuca media (i.e., exceeding the 75^th^ percentile) and chunky medial SMC nuclei (characterized by a length/width ratio below the 25^th^ percentile) were independent risk factors for the development of severe venous graft disease and poor outcome among CABG patients treated with venous aortocoronary bypass grafts. In addition, a low number and marked elongation (a high value for the length/thickness index) of the medial SMC nuclei in the cross sections were associated with a lower prevalence of venous graft disease and more favorable CABG outcomes. The two clinical logistic regression models revealed that only arterial hypertension was an independent risk factor for tunica media hypertrophy but not for a high prevalence of chunky medial SMC nuclei in the cross-sectioned SV segments. Advanced age (>70 years) of the SV graft recipient predicted a low prevalence of histopathological risk factors for SVGD development.

**Table 6:** Results of the three logistic regression models.

Variable	Univariate	Multivariate	OR (95% CI)
**Histopathological model**			
L MedAr	<0.001	0.042	0.19 (0.01–0.83)
H MedAr	0.198	0.394	–
L MedTh	<0.001	0.419	–
H MedTh	<0.001	0.002	3.99 (1.64–9.24)
L Number of medialSMC nuclei	<0.001	0.015	0.23 (0.01–0.76)
H Number of medialSMC nuclei	0.063	0.723	–
L Nucleus length/width	0.003	0.021	1.56 (1.14–3.17)
H Nucleus length/width	<0.001	0.002	0.09 (0.02–0.41)
Clinical model I (risk factors for H MedTh)*			
Age >70 years	<0.001	0.002	0.30 (0.14–0.64)
Gender	0.052	0.101	–
Arterial hypertension	<0.001	0.025	2.29 (1.11–4.76)
Family history of CAD	0.046	0.243	–
Clinical model II (risk factors for L nuclei length/width)*			
Age >70 years	<0.001	0.007	0.32 (0.14–0.73)
Gender	0.161	0.256	–
Arterial hypertension	0.002	0.082	–
Insulin-treated diabetes	0.118	0.206	–
Unstable angina	<0.001	0.705	–
Family history of CAD	0.058	0.168	–

In the clinical models only variables with P<0.2 in the multivariate analysis are presented.

Abbreviations: CAD = coronary artery disease; CI = confidential interval; H = high value (exceeding the 75^th^ percentile) of a given morphometric parameter; L = low value (below the 25^th^ percentile) of a given morphometric parameter; MedAr = tunica media area; MedTh = tunica media thickness; OR = odds ratio; SMC = smooth muscle cell.

## Discussion

The main purpose of the present study was to identify morphometric factors that could predict an accelerated venous graft wall arterialization and, consequently, preterm graft failure. The process of vein arterialization is believed to cause the irreversible reconstruction of the SV graft wall observed months and years after surgery [12]. Although aggressive vein preparation with high-pressure distension may, to some extent, facilitate these processes, it is believed that the biology of the SV transplant is the primary factor predicting these complexities [20]. Early occlusions, recognized up to one month following CABG and involving up to 10–15% of cases, are associated with in-graft thrombosis [12], [21]. Thrombus formation is facilitated by, among other factors, intraoperative damage of SV wall; a discrepancy in the diameter between the larger SV transplant and the recipient coronary artery, resulting in turbulent flow at the site of the distal anastomosis; the poor run-off phenomenon; and perioperative treatment with agents promoting thrombus formation, such as platelet infusions, protamine sulfate or aprotinin administration [9], [16]. It is for these reasons that all patients who developed myocardial infarction during the in-hospital stay or the first month after surgery were not included in the clinical and statistical analysis of late outcomes with respect to histological factors.

The present study revealed variability in the SV wall structure, particularly the histological appearance of the tunica media and SMCs, and this variability had a significant impact on the late outcome after CABG with venous grafts. It was found that in patients with postoperative CAD progression and severe venous graft disease, the SV wall was thicker at the time of the primary surgery due to tunica media thickening. It is not a novel finding that an SV segment with a hypertrophic wall at the time of surgery is a risk factor for late venous graft failure [22]. Previous studies, however, reported that tunica intima hypertrophy followed by the presence of calcium deposits was responsible for the thickening the whole SV wall [23]. These observations are not consistent with our results. In our group, a thick tunica media but not tunica intima was found to be an independent risk factor for venous graft failure. Moreover, we found neither calcium deposits nor poorly differentiated SMCs located subendothelially in the tunica intima. It is highly possible that the absence of these features is a result of the meticulous selection of the venous grafts. It must be stressed again that the SV segments were not obtained from patients with any pathologies of the venous system either at the time of surgery or in the history. Additionally, the use SV segments in patients with symptomatic ischemia of the lower extremities with unacceptable risk of delayed wound healing was avoided. Recently, Hubbell et al. found that chronic hypoxia increases the activity of vascular endothelial growth factor (VEGF), a potent mitogenic molecule for SMCs [24]. In SVGD (+) individuals the values of such histological variables as WallTh and MedTh were comparable to those reported previously as normal values [22], [23]. Thus, it is likely that the SV wall and its media in the SVGD (−) patients was rather hypotrophic compared with that in the SVGD (+) individuals. A hypotrophic SV wall and tunica media might prevent venous graft arterialization. This theory may be supported by the TEM studies, which revealed some differences of potential clinical significance between the medial SMCs observed in the SVGD (−) and SVGD (+) subjects. These cells differed with respect to their intracellular synthetic apparatus. Additionally, in the SVGD (+) group, but not in the SVGD (−) patients, glycogen deposits and substrates for mucopolysaccharide production were observed. In the SVGD (−) patients, the SV segments expressed lipid vacuoles, the possible markers of vascular SMC degeneration [25].

A detailed analysis of the SMCs revealed a significantly higher number of nuclei within the tunica media in CAD (+) patients. Furthermore, elongated SMC nuclei were associated with a better long-term prognosis in CABG patients. We are aware that the analysis of nucleus shape in the cross sections by light microscopy may be affected by marked bias. Thus, in the present study, at least 60 representative nuclei in the tunica media SMCs in the venous segments harvested from each study participant were analyzed. It was noted previously, the population of vascular SMCs was not homogenous and that it was represented by at least two morphological and functional variants that differed with respect to shape, proliferation and chemotactic activity [26], [27], [28]. Moreover, the SMC nucleus shape *per se* may influence DNA synthesis and, as a consequence, cellular activity [29]. Li et al. found that PDGF, a potent mitogenic molecule, is active exclusively in the epithelioid-like SMCs isolated from the human internal mammary artery but not in the spindle-like cells [30]. Interestingly, elongated cells are usually numerous in the tunica media of the intact vessel wall, whereas endothelial-like cells are present in the areas of previous damage to the intima including areas that experienced surgical and procedural trauma [27], [31]. Thus, our findings regarding the shape of SMC nuclei, support the hypothesis that the morphology of these nuclei could be regarded as a potential predictor of the SV graft patency rate and the long-term CABG outcomes.

The results of our study support earlier reports indicating that postoperative clinical CAD deterioration is related to both SV graft failure and the progression of atherosclerosis in the native coronary arteries [32], [33]. It was discovered that a majority of CABG individuals with postoperative CAD clinical progression had lesions compromising blood flow in the SV grafts. In fewer than 50% of these cases, these lesions were accompanied by disease progression in the either grafted or ungrafted native coronary arteries. The latter phenomenon was observed commonly in diabetic patients treated with insulin. The comparison of the SVGD (−) and SVGD (+) patients in the present study revealed some clinical variables of potential interest in predicting CABG outcomes. These variables are accepted risk factors for atherosclerosis development and progression [4], [11], [17]. However, the “clinical” multivariate logistic regression models, commonly used to identify independent risk factors, revealed that among the demographic and clinical variables only age (above 70 years) and arterial hypertension might have impact on the CABG outcomes. We are aware that the enrollment of more patients would most likely allow the identification of more clinical determinants of both SVGD and the accelerated progression of the disease in the native coronary vessels. We would like to stress again that the assessment of clinical predictors of SVGD was not the primary purpose of this study.

The findings of this study may be of great interest in the management of CAD patients undergoing CABG. It was shown that making a decision regarding whether to use SV segments only on the basis of the macroscopic graft appearance is not sufficient. We demonstrated some of the SV segments assessed intraoperatively as transplants of adequate quality were in practice not optimal for use as aortocoronary grafts in terms of the long-term outcome.

### Conclusions

The ultrastructural appearance of the saphenous vein tunica media and the medial smooth muscle cells may determine the fate of venous aortocoronary bypass grafts and the late CABG outcomes. Thickening of the venous wall related to tunica media hypertrophy and smooth muscle cell hyperplasia, followed by the increased appearance of synthetic structures in these cells, may predict accelerated venous graft failure.
